# Further spreading of canine oriental eyeworm in Europe: first report of *Thelazia callipaeda* in Romania

**DOI:** 10.1186/s13071-015-0663-2

**Published:** 2015-01-27

**Authors:** Andrei Daniel Mihalca, Gianluca D’Amico, Iuliu Scurtu, Ramona Chirilă, Ioana Adriana Matei, Angela Monica Ionică

**Affiliations:** Department of Parasitology and Parasitic Diseases, University of Agricultural Sciences and Veterinary Medicine Cluj-Napoca, Faculty of Veterinary Medicine, Calea Mănăștur 3-5, Cluj-Napoca, 400372 Romania; Department of Internal Medicine, University of Agricultural Sciences and Veterinary Medicine Cluj-Napoca, Faculty of Veterinary Medicine, Calea Mănăștur 3-5, Cluj-Napoca, 400372 Romania; Faculty of Environmental Protection, University of Oradea, Strada General Magheru, Oradea, 410048 Romania

**Keywords:** *Thelazia callipaeda*, Emerging disease, Canine vector-borne diseases

## Abstract

**Background:**

Despite the increasing number of reports of autochthonous cases of ocular thelaziosis in dogs in several European countries, and the evident emergence of human cases, the distribution and spreading potential of this parasite is far for being fully known. In Romania, despite intensive surveillance performed over recent years on the typical hosts of *T. callipaeda*, the parasite has not been found until now.

**Methods:**

In October 2014 a German Shepherd was presented for consultation to a private veterinary practice from western Romania with a history of unilateral chronic conjunctivitis. Following a close examination of the affected eye, nematodes were noticed in the conjunctival sac. The specimens collected were used for morphological examination (light microscopy) and molecular analysis (amplification of the cytochrome *c* oxidase subunit 1 gene, followed by sequencing).

**Results:**

Thirteen nematodes were collected, all identified morphologically as *T. callipaeda*. The history of the dog revealed no travel outside Romania, and during the last year, not even outside the home locality. The BLAST analysis of our sequence showed a 100% similarity *T. callipaeda* haplotype h1.

**Conclusions:**

This is the first report of *T. callipaeda* in Romania, which we consider to be with autochthonous transmission. These findings confirm the spreading trend of *T callipaeda* and the increased risk of emerging vector-borne zoonoses.

## Background

*Thelazia callipaeda* is a vector-borne zoonotic eyeworm, parasitizing the conjunctival sac of domestic and wild carnivores (foxes, beech martens and wolves), rabbits and humans. Its presence is associated with mild to severe ocular disease [1,2]. The distribution includes vast territories in Asia (hence the name “oriental eye worm”) but also in former Soviet Union [3]. In Europe, the first report came from Italy [4] followed by various subsequent records in the same country [5-7]. However, during the last decade, the knowledge about its distribution in Europe has been greatly expanded (Table 1). The vector was demonstrated by Otranto *et al.* [8,9], when the nematodes were successfully transmitted by the drosophilid fly, *Phortica variegata* (Drosophilidae, Steganinae).

**Table 1:** **Emergence of** ***Thelazia callipaeda*** **in Europe between 2007 and 2014**

**Year**	**First report of autochthonous cases**	**Reference**
2007	France	[10]
2008	Switzerland	[11]
2010	Germany	[12]
2011	Spain	[13]
2012	Portugal	[14]
2014	Bosnia and Herzegovina	[2]
2014	Croatia	[2]
2014	Romania	Present study

All the *T. callipaeda* isolates in Europe for which sequences of partial cytochrome c oxidase subunit 1 (*cox*1) are available belong to the haplotype 1 (h1), suggesting a high degree of nematode-host affiliations for this haplotype [15].

Based on climatic analysis, a wider European distribution was suggested already in 2003 by Otranto *et al.* [7]. Despite our recent intensive surveillance on vector-borne diseases of wild (foxes, jackals, wolves, wild cats, lynxes) and domestic carnivores (dogs, cats) in Romania [16-24], until now we were not able to confirm the presence of this zoonotic helminth in Romania.

The aim of this study was to extend the knowledge on the geographical distribution of *T. callipaeda* in Europe and to identify the haplotype circulating in Romania.

## Methods

In October 2014, a dog (German Shepherd x Siberian Husky cross breed, castrated male, 9 years old) was presented for consultation to a private veterinary practice from Oradea, Bihor County, in western Romania (47.06 N, 21.90E) with a history of unilateral chronic conjunctivitis (right eye). After one month of local intra-conjunctival treatment with antibiotics, as the animal’s condition was not improving, the owner brought the case to our attention (by author RC). Following a close examination of the affected eye, alive, white, medium-sized nematodes were noticed in the conjunctival sac. As part of the treatment, all nematodes were collected during superficial anaesthesia (Xylazine + Ketamine), using a fine blunt tweezers and preserved for further examination in absolute ethanol (3 specimens) and 5% formalin (10 specimens). We have obtained the verbal consent of the owner to use the collected material for a scientific publication and he kindly provided the travel history of the dog.

The specimens collected in formalin were used for morphological examination. The nematodes were mounted on a glass slide, cleared with lactophenol and examined using an Olympus BX61 microscope. Photographs and measurements for morphologic identification were taken using a DP72 camera and Cell^F software (Olympus Corporation, Japan).

The specimens collected in absolute ethanol were analysed using molecular techniques. Genomic DNA was extracted from a gravid female using a commercial kit (Isolate II Genomic DNA Kit, BIOLINE, UK) according to the manufacturer’s instructions. Amplification of a partial cytochrome *c* oxidase subunit 1 (*cox*1) gene of spirurid nematodes (670 bp) was performed using the NTF/NTR primer pair, following reaction procedures and protocols described in literature [25]. PCR products were visualized by electrophoresis in a 2% agarose gel stained with RedSafeTM 20000× Nucleic Acid Staining Solution (Chembio, UK) and their molecular weight was assessed by comparison to a molecular marker (O’GeneRulerTM 100 bp DNA Ladder, Thermo Fisher Scientific Inc., USA). Amplicons were purified using silica-membrane spin columns (QIAquick PCR Purification Kit, Quiagen) and then sequenced (performed at Macrogen Europe, Amsterdam). Sequences were compared to those available in GenBank™ dataset by Basic Local Alignment Search Tool (BLAST) analysis.

## Results

**Figure 1:**
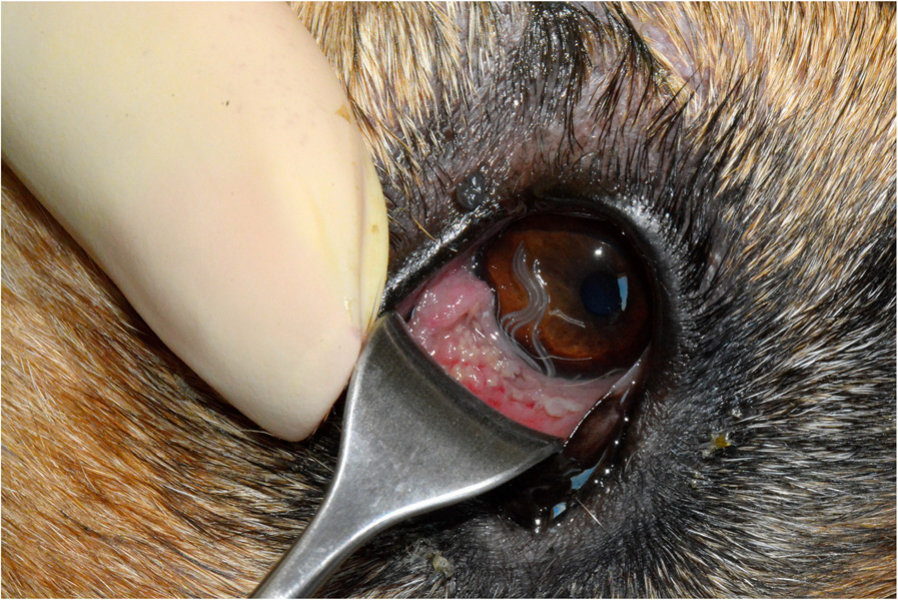
**Clinical aspect of the infection with the presence of nematodes.**

From the conjunctival sac of the right eye, 12 nematodes were collected. Additionally, one nematode was also found in the conjunctival sac of the apparently non-affected left eye. A close examination of the affected right eye revealed the presence of proliferative lesions in the inferior conjunctival sac (Figure 1), epiphora and conjunctivitis. The history of the dog as recalled by the owner did not include any travel outside Romania, and in the last year, not even outside the city limits of Oradea.

**Figure 2:**
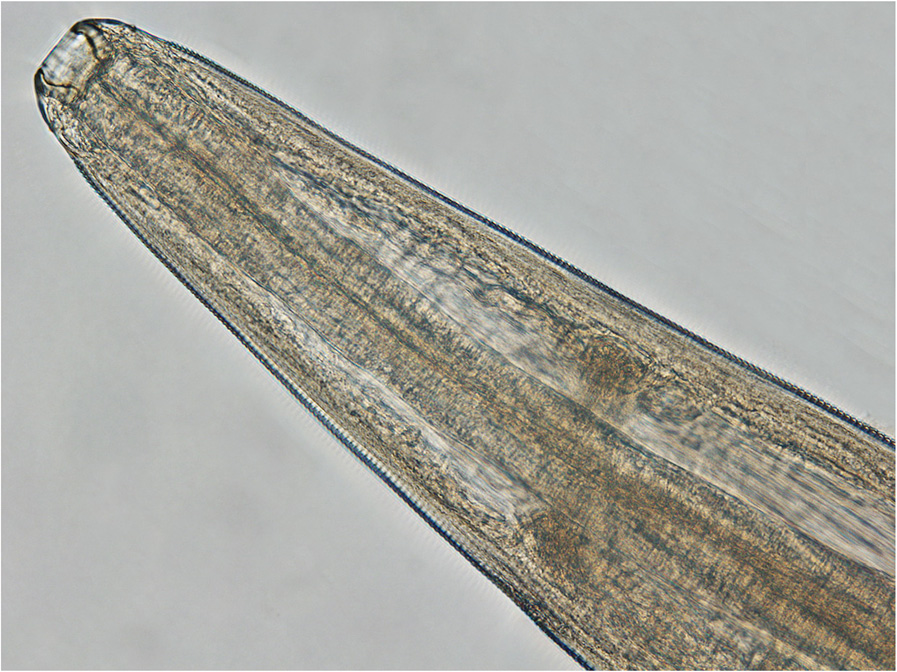
**Typical morphology of the anterior extremity of female** ***T. callipaeda*** **(x400).**

Light microscopy (Figure 2) examination of the nematodes revealed typical specific features of *T. callipaeda* [26]. All 13 collected nematodes were females (no males were found).

The BLAST analysis of our sequence (GenBank™ accession number KP087796) showed a 100% similarity to a sequence (GenBank™ accession number AM042549) of *T. callipaeda* haplotype h1 [15].

## Discussion

After the previous records in Europe (Italy, France, Switzerland, Germany, Spain, Portugal, Bosnia and Herzegovina, Croatia), the current study reports the presence of the zoonotic eyeworm *T. callipaeda* for the first time in Romania. Considering that the host dog has never travel to other known endemic areas, nor outside the city limits in the last year, we regard this as a sufficient proof for the existence of an autochthonous transmission cycle. So far, this is the most easternmost report in Europe (except previous records from the former USSR), confirming in our opinion the spread of this nematode.

Often, disease emergence and spreading, is only apparent due to lack of sufficient investigation, mainly in the case of non-clinical infections which require targeted laboratory diagnosis. However, in the case of canine ocular thelaziasis, we consider the disease new to Romania, as the infection is usually clinical and can be hardly overlooked by owners and clinicians. Moreover, in the past 5 years, the authors of the present paper had intensively focused on the surveillance of vector-borne pathogens in domestic and wild carnivores, with more than one thousand potential hosts individuals investigated and specifically examined for eye worms from various regions of the country (including western Romania). Additionally, to our knowledge, *T. callipaeda* was not found to date in any of the neighbouring countries (i.e. Hungary, Bulgaria, Serbia, Republic of Moldavia or Ukraine).

The only confirmed vector for *T. callipaeda* is *Phortica variegata* (Diptera, Drosophilidae, Steganinae) has been reported in Romania on various occasions [27]. Its presence is known from the following counties: Buzău, Giurgiu, Constanța, Caraș-Severin, Mehedinți, Timiș, Maramureș, Ialomița and Teleorman [27]. As Oradea (Bihor County) has similar climatic and ecologic conditions with the known area of *P. variegata* occurrence in Romania, the vector is also probably present here. However, further entomological surveys are required for its confirmation.

Genetic analysis of *cox*1 confirmed the existence in Europe of a single haplotype, as defined earlier [15], suggesting a west to east spread of the parasite in Europe. However, it is not clear which are the possible routes of disease spreading, but most probably this is related to host circulation rather that vector emergence or climate change.

Cats have been also implicated in clinical cases of ocular infections with *T. callipaeda*, with reports from Italy, France, Portugal and Switzerland [5,7,10,28-31]. Recent data suggest also the potential reservoir role of wildlife in natural transmission cycles of this spirurid [32-34].

## Conclusion

As *T. callipaeda* is an emerging zoonotic infection [35], our findings bring new important epidemiological data highlighting the need for increased awareness among owners, veterinarians and ophthalmologists, even outside the known endemic areas.
